# Editorial: Endophytic fungi producers of biomolecules of interest to human health

**DOI:** 10.3389/ffunb.2024.1239470

**Published:** 2024-01-30

**Authors:** Pérola O. Magalhães, João Vicente Braga de Souza, Danilo Batista Pinho

**Affiliations:** ^1^Department of Pharmacy, Health Sciences School, University of Brasilia, Brasilia, Brazil; ^2^Mycology Laboratory, National Institute for Amazonian Research (INPA), Manaus, Brazil; ^3^Department of Phytopatology, University of Brasilia, Brasilia, Brazil

**Keywords:** endophytic fungi, biomolecules, human health, bioprocess, eukaryotic organism

Fungi are eukaryotic organisms that inhabit living plants and animals or dead tissues. They are important sources of food, alcohol, enzymes, antibiotics, organic acids, and plant growth-promoting compounds. Endophytic fungi residing inside the aerial tissues of plants do not produce any apparent harm to the host plant (Khan et al., 2023). Instead, they promote plant growth in a variety of ways, including through the release of plant growth hormones, such as cytokines, indole acetic acid, or gibberellins, and by providing biologically fixed nitrogen (Bilal et al., 2018). These fungi have been studied for their role in improving plant protection, in addition to being important sources of new molecules for the development of new drugs.

The research on “*Endophytic fungi producers of biomolecules of interest to human health*” is important to our team to help foster knowledge of these fungi. It includes three original research articles written by scientists from Brazil and Germany. The team thanks all of the researchers involved in this work for their support and the results.

Two papers focused on endophytic fungi isolated from *Bauhinia variegata* leaves. In one of these, seven fungi that have antioxidant activities were identified. One of the extracts presented pan-agonist activity in peroxisome proliferator-activated receptors (PPARs), while another showed activity in α, β/δ, and γ (Mesquita et al.). The other paper studied the antioxidant activity of the mycelial methanolic extracts of two endophytic fungi and their action as PPAR agonists. Moreover, their effect on the activity of antioxidant defense system enzymes was evaluated (Pires et al.).

Lastly, the third article, whose authors are from Germany, presented genome mining as a biotechnological tool for the discovery of novel biosynthetic genes in lichens (Singh et al.).

Therefore, the articles published as part of the Research Topic “*Endophytic fungi producing biomolecules of interest to human health*” put forward interesting and current information on the proposed topic.

## Author contributions

All authors listed have made a substantial, direct, and intellectual contribution to the work and approved it for publication.
